# Knowledge, attitudes, and practices toward leishmaniasis and one health: a cross-sectional study among medical and veterinary professionals

**DOI:** 10.3389/fvets.2024.1515370

**Published:** 2025-01-24

**Authors:** Yasir Khan, I-Chen Lin, Sundus Khan, Mehtab Kanwal, Abdul Wajid, Aamir Khan, Fazal Noor, Ali Madi Almajwal, Chien-Chin Chen, Abdul Qadeer

**Affiliations:** ^1^Lady Reading Hospital Peshawar, Peshawar, Pakistan; ^2^Division of Colorectal Surgery, Department of Surgery, Ditmanson Medical Foundation Chia-Yi Christian Hospital, Chiayi, Taiwan; ^3^Kuwait Teaching Hospital Peshawar, Peshawar, Pakistan; ^4^Institute of Zoological Sciences, University of Peshawar, Peshawar, Pakistan; ^5^Faculty of Pharmacy, Gomal University, Dera Ismail Khan, Khyber Pakhtunkhwa, Pakistan; ^6^Livestock and Dairy Development Department (Extension Wing), Khyber Pakhtunkhwa, Pakistan; ^7^Livestock and Dairy Development Department (Research Wing), Khyber Pakhtunkhwa, Pakistan; ^8^Department of Community Health Sciences, College of Applied Medical Sciences, King Saud University, Riyadh, Saudi Arabia; ^9^Department of Pathology, Ditmanson Medical Foundation Chia-Yi Christian Hospital, Chiayi, Taiwan; ^10^Department of Cosmetic Science, Chia Nan University of Pharmacy and Science, Tainan, Taiwan; ^11^Doctoral Program in Translational Medicine, National Chung Hsing University, Taichung, Taiwan; ^12^Department of Biotechnology and Bioindustry Sciences, College of Bioscience and Biotechnology, National Cheng Kung University, Tainan, Taiwan; ^13^Department of Cell Biology, School of Life Sciences, Central South University, Changsha, China

**Keywords:** leishmaniasis, one health, zoonotic, global health, attitude

## Abstract

Leishmaniasis is a significant zoonotic infection with global health implications, particularly in regions where human and animal health are closely interconnected. This cross-sectional study assessed the knowledge, attitudes, and practices (KAP) of 5,074 participants regarding leishmaniasis and the One Health concept. The socio-demographic data revealed that most respondents were young (82.6%), male (82.3%), and from rural areas (50.8%), with a majority based in Khyber Pakhtunkhwa (57.4%). Veterinary professionals (42.1%) and students (27.4%) constituted the primary respondents, with 32.4% working in government hospitals. Knowledge about leishmaniasis was high, with 97.5% of participants recognizing Leishmania and 86% correctly identifying it as a protozoan disease. The majority (71.8%) believed in the zoonotic transmission of Leishmania from animals to humans. Attitudes toward the One Health concept were positive, with 90.2% of respondents aware of it, and 95.5% acknowledged the zoonotic nature of the disease. Practices for controlling sandfly populations were observed by 56.4% of participants, with bed nets (44.9%) being the most common preventive measure. Results showed that younger participants had significantly better knowledge, attitude, and perception regarding leishmaniasis and One Health compared to older individuals. Veterinarians and government hospital staff demonstrated better KAP toward VL. This study underscores the importance of educational interventions and community-based control measures to enhance understanding and prevention of leishmaniasis, with the One Health approach playing a crucial role.

## 1 Introduction

Leishmaniasis is a major neglected tropical infectious disease caused by protozoan parasites, ranking as the ninth leading contributor to the global burden of infectious diseases, posing a significant threat to public health worldwide (1). The causative parasites belong to the genus Leishmania, within the family Trypanosomatidae. Transmission occurs via the bites of infected female sandflies, specifically from the genus Phlebotomus in the Old World and Lutzomyia in the New World. Currently, there are at least 93 identified sandfly species recognized as confirmed or likely vectors on a global scale (2, 3). The disease has progressively expanded, spreading across diverse geographical regions, infecting multiple hosts, and involving numerous vector species (4). To date, approximately 23 Leishmania species are known to be responsible for human infections (5).

The genus Leishmania exhibits considerable diversity and follows a complex life cycle with two major developmental stages: the promastigote, which exists in the invertebrate vector (sandfly), and the amastigote, which occurs in the vertebrate host, such as humans or animals. More than 20 species of Leishmania are implicated in human infections (5). Once introduced into the host’s dermis by the sandfly, the parasite circumvents the host’s immune defenses by invading and proliferating within phagocytic cells, primarily macrophages. This intracellular survival leads to a spectrum of clinical manifestations, each associated with distinct prognoses (6, 7).

Leishmaniasis manifests in four clinical forms: visceral leishmaniasis (VL), cutaneous leishmaniasis (CL), mucocutaneous leishmaniasis, and post-kala-azar dermal leishmaniasis (PKDL). The disease is endemic in many tropical and subtropical regions, affecting over 12 million people across at least 88 countries. It contributes to a global disease burden of 2.4 million disability-adjusted life years (DALYs) and causes approximately 70,000 deaths annually (8–10). According to the World Health Organization (WHO), the annual incidence of CL ranges from 600,000 to 1 million cases, while VL accounts for 50,000 to 90,000 cases each year. Around 90% of cases for both forms are reported in different countries, such as Afghanistan, Bangladesh, Brazil, Saudi Arabia, Syria, and Peru (11). Individuals often become infected when exposed to endemic areas, and more than 1 billion people worldwide are considered at risk of contracting the disease (12). CL represents a significant public health challenge in these regions, as its diverse epidemiological characteristics and clinical presentations complicate disease control (13). While VL remains a fatal condition, CL is the most widespread form of the disease globally (14).

Leishmaniasis is a significant public health concern in Pakistan, affecting both human and animal reservoirs (1). It ranks as the second most prevalent vector-borne disease after malaria in the country (15). Annually, between 21,700 and 35,700 cases of cutaneous leishmaniasis (CL) are reported, with outbreaks frequently occurring in regions of Pakistan such as Punjab (Multan), Baluchistan, and Khyber Pakhtunkhwa (16). The disease disproportionately impacts low-income and marginalized communities due to limited access to healthcare services (17). The leishmaniasis situation in Pakistan has worsened over time, with the interior areas of Sindh, Khyber Pakhtunkhwa, and Baluchistan identified as endemic for Leishmania tropica, the most encountered species in the country (18). The epidemiology of leishmaniasis exhibits significant dynamism, with transmission conditions continually evolving due to factors such as demography, environmental changes, human behavior, and the immunological profiles of the impacted populations (19). The control measures vary significantly because of the wide range of Leishmania species, biological factors, and different reservoir hosts. In addition to these factors, housing, low socioeconomic conditions, and interaction with pets were found to be linked to a heightened risk for cutaneous leishmaniasis (20).

The One Health approach emphasizes the interconnectedness of human animal health, and the environment (21). The One Health approach addresses zoonotic diseases by promoting health and well-being through the prevention and mitigation of disease risks at the interface of animals, humans, and the environment (22). Over the past few decades, numerous studies have explored various modifiable and non-modifiable risk factors. However, no study has yet examined the knowledge and application of the One Health approach to leishmaniasis control and prevention among healthcare professionals, including medical doctors, veterinary doctors, paramedics, Paravet staff, and medical and veterinary students. The primary objective of this study was to assess the knowledge, attitude, and perception of medical professionals in Pakistan concerning leishmaniasis and the One Health concept.

## 2 Materials and methods

### 2.1 Study design

A web-based questionnaire was designed to collect data for this cross-sectional study. Based on the objectives of the study, a target population of Pakistan’s medical or veterinary profession was approached through a web-based survey from July 01 to July 30, 2024. We considered a response distribution of 5% and a margin of error of 95% to determine the required sample size. Thus, the sample size was approximately 385 respondents however, to get more exact and accurate results, we collected 5,163 responses for the survey.

### 2.2 Data collection tool

We conducted an online survey to collect data. Cronbach’s Alpha test was utilized to evaluate the reliability of the knowledge regarding leishmania, one health principles, risk factors attitude, and perceptions about the association between one health knowledge and risk factors questions independently. The value of Cronbach’s Alpha for knowledge is 0.75, and attitude is 0.71 and 0.67 for perception. It shows that knowledge questions have stronger internal consistency than Attitudes and Perceptions. A snowball sampling method was used to select participants, and one email address was permitted per respondent. The participants were advised to complete and submit their responses using a computer or a mobile device. There were four sections in the questionnaire. Data on socio-demographic characteristics were included in the first part, in addition to gender, age, Marital status, Residence, Administrative Units, education, and employment status. The second section contained 13 multiple-choice questions designed to measure knowledge. The third section had 8 attitude-testing questions, while the fourth contained 11 questions to evaluate participant perception regarding the association between one health knowledge and risk factors. “Yes,” “No,” “Not sure,” and multiple-choice response options were given to respondents for each question. The survey was written in English.

### 2.3 Data collection procedure

The survey did not include personal information like name, addresses, etc., that identify the respondent. To complete a structured questionnaire, a specific platform link, and a Google form were created and circulated through social media. It was shared with different groups on WhatsApp and Facebook, and the admin and members of these groups were requested to share the link to get enough responses. Before starting the replies, each respondent was asked to confirm that they had informed permission by clicking the consent declaration. “I do with this, after reading the aims of the study, engage in the survey supplying my information by answering questions rationally and willingly,” was the informed consent statement offered to the respondents. Respondents filled out the survey and clicked the “submit” button to send it to our platform for data collection. To confirm the authenticity of the responses, all questions were compulsory to be answered.

### 2.4 Study variables

A total of 32 questions with multiple response options were used to assess knowledge about leishmania, one health principles, and perceptions about the association between one health and risk factors. The knowledge score was 0 (lowest) to 10 (highest). Age, which was divided into groups of 20 to 35 years, 36 to 50 years, and 51 to 65 years; gender (male or female); marital status (single or married); participants’ residency area of Pakistan (Khyber Pakhtunkhwa, Sindh, Azad Kashmir, Islamabad Capital Territory, Punjab, Baluchistan, and Gilgit-Baltistan); and urban or rural area. Furthermore, participants were asked about occupation, and education was divided into five levels: Bachelor Enrolled (4th / 5th year of degree), Doctorate, Master, and Diploma postgraduate. Thirteen questions focused on perception, and 19 on attitude and practice in anticipating the disease.

### 2.5 Statistical analysis

The responses collected via Google Forms were exported to Microsoft Excel. Statistical analysis was conducted in SPSS version 2023. The variables were coded in SPSS, and missing values were checked using Missing Value Analysis. Furthermore, a new variable was created in SPSS by applying a filtering condition using the “IF” function. This condition excluded the responses from individuals who either had not heard of Leishmania, or One Health or did not have pets. The same filtering logic was applied to other responses where specific conditions were imposed. Factors linked with Leishmania knowledge, attitude, and perception were discovered using chi-square. Descriptive statistics such as frequency and percentage were employed to demonstrate the demographic features of the data. The chi-square test was carried out to evaluate response variables and explanatory factors. A *p*-value of 0.05 was set to determine statistical significance.

## 3 Results

### 3.1 A socio-demographic variable of the respondents

A total of 5,074 individuals participated in this study. In this survey, most of the participants (82.6%) were young. Most respondents were male, 82.3%, single (55.8%), and (50.8%) were from rural areas. Among the participants, most were from Khyber Pakhtunkhwa province (57.4%). The data collected from different professional people shows most of the participants were Veterinary doctors (42.1%) followed by veterinary/medical students (27.4%). Most of the respondents of this survey were found to be enrolled in bachelor’s degree (4^th^/5^th^ year) (27.9%) and in-service (53.4%) working in Government hospitals (32.4%). Table 1 provides details of the demographic characteristics of the studied participants.

**Table 1:** Socio-demographic variables of participants (*N* = 5,074).

S. no	Variable	Unique values	Frequency	Percentage (%)
1	Age	20–35	4,191	82.6
		36–50	712	14
		51–65	72	1.4
		Do not want to share	99	1.9
2	Gender	Male	4,178	82.3
		Female	896	17.4
3	Marital Status	Single	2,832	55.8
		Married	2,214	43.6
		Do not want to share	28	0.5
4	Administrative Unit	Khyber Pakhtunkhwa	2,915	57.4
		Punjab	696	13.7
		Balochistan	648	12.8
		Sindh	351	6.9
		Azad Jammu Kashmir	128	2.5
		Gilgit Baltistan	112	2.2
		Islamabad Capital Territory	224	4.3
5	Occupation	Medical doctor	771	15.2
		Veterinary Doctor	2,138	42.1
		Paramedic staff	430	8.5
		Paravet staff	344	6.8
		Student (Medical / Veterinary)	1,391	27.4
6	Education	Diploma / Intermediate	740	14.6
		Bachelor enrolled (4^th^ / 5^th^ of degree)	1,415	27.9
		Bachelor	933	18.4
		Master	1,326	26.1
		Doctorate	474	9.3
		Post-doctorate	186	3.7
7	Residence	Urban	2,498	49.2
		Rural	2,576	50.8
8	Employment Status	Retired	30	0.6
		Inservice	2,707	53.4
		Unemployed	906	17.9
		House job / internship	592	11.7
9	Workplace	Government hospital	1,644	32.4
		Private hospital	260	5.1
		University	840	16.6
		Private sector (industry/farm/clinic/NGO)	459	9.0
		College	48	0.9
		Research Institute	267	5.3
		N/A	1,556	30.7

### 3.2 Knowledge regarding leishmaniasis

Table 2 briefly explains the knowledge of participants regarding VL. In this survey, out of the 5,074 participants, 4,949 (97.5%) affirmed that they had been knowledgeable about Leishmania. One hundred twenty-five participants (2.5%) indicated that they did not know about Leishmania, which was excluded from further adding their responses to the questions that followed by generating a function of “if” in SPSS. Out of these 4,949 knowledgeable respondents, a total of 4,255 (86%) gave positive responses regarding the causative agent in which they opted for protozoa as the causative agent, 163 (3.3%) participants stated that bacteria could cause it, 118 participants (2.4%) confirmed the virus as a causative agent, 128 (2.6%) choose fungus, and 216 (4.4%) participants assured Helminths as a causative agent. There is remaining 69 (1.4%) opted, unaware of the causative agent option. Overall, 2,113 (42.7%) participants recorded Leishmania as infectious, as well as contagious, while 2,450 respondent considered it as non-infectious and slightly more respondents (2455) claimed Leishmania as non-contagious. In addition, 2,376 participants (48%) were confident that leishmania could be transmitted between humans and humans, and 3,220 (65.1%) participants recognized their transmission from animals to animals. Concerning leishmania transmission between humans and animals and vice-versa, most participants 3,553 (71.8%) believed their transmission from animals to humans. In comparison, 1,009 (20.4%) did not believe their transmission, and 1925 (38.9%) were assured about the transmission of leishmania from human to animal, while 1970 participants (39.8%) disagreed their transmission. In addition, 2,515 (50.8%) participants also stated the vertical transmission of Leishmania, and most of the participants, 2,519 (50.9%) disagreed with the transmission of Leishmania from infected animal’s milk and meat, followed by 1,504 (30.4%) participants who recognized the horizontal transmission of Leishmania. In terms of medical treatment, most of the respondents, 4,340 (87.7%), were sure about their treatment, and out of these, 3,454 (79.6%) of participants affirmed Antiprotozoal use as the drug of choice for its treatment.

**Table 2:** Participants knowledge related to leishmaniasis (*N* = 5,074).

S. no	Question / variable	Value	Frequency	Percentage
1	Have you heard about Leishmania?	Yes	4,949	97.5
		No	125	2.5
2	What is the causative agent of Leishmania?	Bacteria	163	3.3
		Virus	118	2.4
		Fungus	128	2.6
		Protozoa	4,255	86.0
		Helminths	216	4.4
		Do not Know	69	1.4
3	Is Leishmania an infectious disease?	Yes	2,113	42.7
		No	2,450	49.5
		Do not Know	386	7.8
4	Is Leishmania Contagious?	Yes	2,113	42.7
		No	2,455	49.6
		Do not Know	381	7.7
5	Do you hear about mortality from leishmania?	Yes	2,732	55.2
		No	1821	36.8
		Do not Know	396	8.0
6	Is leishmania transmitted from human to human?	Yes	2,376	48.0
		No	1950	39.4
		Do not Know	623	12.6
7	Is leishmania transmitted from animal to animal?	Yes	3,220	65.1
		No	1,231	24.9
		Do not Know	498	10.0
8	Is leishmania transmitted from animal to human?	Yes	3,553	71.8
		No	1,009	20.4
		Do not Know	387	7.8
9	Is leishmania transmitted from human to animal?	Yes	1925	38.9
		No	1970	39.8
		Do not Know	1,054	21.3
10	Does leishmania transmit from pregnant women to their offspring?	Yes	2,515	50.8
		No	1,568	31.7
		Do not Know	866	17.5
11	Does leishmania transmit from infected animals’ meat or milk?	Yes	1,504	30.4
		No	2,519	50.9
		Do not Know	926	18.7
12	Have you ever heard about leishmania treatment?	Yes	4,340	87.7
		No	371	7.5
		Do not Know	238	4.8
13	If yes, what treatment is done for leishmania infection?	Antibiotic	664	15.3
		Antiviral	74	1.7
		Antiprotozoal	3,454	79.6
		Anthelminthic	148	3.4

### 3.3 Attitude toward leishmaniasis and one health

Table 3 presents respondents’ attitudes regarding VL and One Health. The data recorded shows that the majority of 4,576 (90.2%) of the participants knew about One Health, and 9.8% had no knowledge about One Health. Out of these 4,576, most of the participants, 1864 (40.7%), considered one health as “Human health is connected to animal health. The majority of participants, 3,661 (80%), considered animal health very important when addressing human health issues. Furthermore, most of the participants, 2,448 (53.5%), claimed that they had received formal education on “One Health Concept,” while the remaining did not receive any formal education. In this survey, we found that among 4,949 Leishmania knowledgeable respondents, about 4,726 (95.5%) participants had positive knowledge about zoonosis, and 4.5% were not aware of zoonosis. Out of these 4,726 participants, 4,206 (89%) knew that Leishmania was a zoonotic infection. Regarding the transmission of the parasite, out of 4,949 Leishmania knowledgeable participants, 76.7% of respondents recorded that it is through sand flies. In this study, most of the participants, 71.6%, stated that Leishmania is not reported in their household, while the remaining participants reported their presence.

**Table 3:** Attitude toward leishmania and one health.

S. no	Question / variable	Value	Frequency	Percentage
1	Have you heard of the term “One Health”?	Yes	4,576	90.2
		No	498	9.8
2	If yes, what does “One Health” mean to you? (Select all that apply)	Human health is connected to animal health	1864	40.7
		Environmental factors affect health	1,622	35.5
		Collaboration between various health disciplines	1,090	23.8
3	How important is it to consider animal health when addressing human health issues?	Very Important	3,661	80.0
		Important	755	16.5
		Moderately important	82	1.8
		Slightly important	78	1.7
4	Have you received any formal education or training on One Health concepts?	Yes	2,448	53.5
		No	2,128	46.5
5	Do you know Zoonosis?	Yes	4,726	95.5
		No	223	4.5
6	Is Leishmania a Zoonosis disease?	Yes	4,206	89.0
		No	520	11.0
7	Do you have any idea about leishmania transmission?	Water	173	3.5
		Livestock	356	7.2
		Sandflies	3,796	76.7
		Insects	520	10.5
		Soil	104	2.1
8	Have you or anyone in your household ever been diagnosed with leishmaniasis?	Yes	1,406	28.4
		No	3,543	71.6

### 3.4 Participants practices and perception toward CL prevention and control

Table 4 revealed that out of 5,074 participants, 3,422 (67.4%) participants have reared animals/pets, among which 32.6% have reared dogs. Approximately 39.7% of participants claimed that their animals stayed indoors. In addition, 56.4% of participants claimed that various measures were taken in the community for the control of sandfly populations, among which 27.7% of participants observed insecticide spraying, 44.9% used bed nets, and 14.7% observed environmental management, i.e., clearing vegetation, while about 45.8% participants were using bed nets daily. Approximately 74.6% of participants recognized that knowledge about One Health can help prevent leishmaniasis, and 6% did not agree, while 19.4% of participants were not sure about the role of One Health knowledge in preventing leishmaniasis. The majority of the participants had implemented preventive measures in their homes and communities based on one health knowledge, while the remaining had not adopted any measures.

**Table 4:** Practices and perception toward control and prevention of leishmaniasis in study participants (*N* = 5,074).

S. no	Question / variable	Value	Frequency	Percentage
1	Do you have rear animals or pets?	Yes	3,422	67.4
		No	1,652	32.6
2	If yes, which types? (Select all that apply)	Dogs	1,116	32.6
		Cats	339	9.9
		Cattle	905	26.4
		Goats	452	13.2
		Mixed (Cats, Cattle, Goats and Sheep)	124	3.6
		Others	486	14.3
3	Do your animals stay indoors, outdoors, or both?	Indoors	1,359	39.7
		Outdoors	643	18.8
		Both	1,420	41.5
4	Are there measures taken in your community to control sandfly populations?	Yes	2,862	56.4
		No	2,212	43.6
5	If yes, what measures? (Select all that apply)	Insecticide spraying	793	27.7
		Use of bed nets	1,285	44.9
		Environmental management (e.g., clearing vegetation)	421	14.7
		Others	363	12.7
6	Do you use bed nets daily?	Yes	589	45.8
		No	696	54.2
7	Do you think having knowledge about One Health can help in preventing leishmaniasis?	Yes	3,414	74.6
		No	275	6.0
		Maybe	887	19.4
8	Have you implemented any preventive measures in your home or community based on your knowledge of One Health?	Yes	3,501	76.5
		No	1,075	23.5

### 3.5 Association of knowledge, attitude, and perception with the demographic variables

In this study, we checked the association of knowledge, attitude, and perception with demographic variables like age, gender, residence, administrative unit, occupation/profession, education, and workplace. The comprehensive results of the regression models including demographic parameters are given in (Supplementary Tables 1–7). In Supplementary Table 1, the relationship between knowledge, attitude, and perception with the demographic variable age showed a statistically significant correlation, indicating that younger individuals tend to possess a greater understanding of Leishmania. It was also observed that a younger age correlates with enhanced knowledge. An unfavorable attitude was notably linked to older age, whereas younger individuals exhibited a more positive attitude toward Leishmania and the concept of one health. Similarly, a positive perception was observed among younger ones toward Leishmania and one health in comparison to older ones. In Supplementary Table 2, it is observed that inadequate knowledge, attitude, and perception were significantly linked to women in comparison to men. The distribution of knowledge, attitude, and perception levels varied significantly between urban and rural areas. Interestingly, it was observed that the levels of knowledge, attitude, and perception were somewhat greater in rural areas than in urban areas (Supplementary Table 3). The distribution of knowledge, attitude, and perception levels varied significantly across the provinces and administrative units. Positive knowledge, attitude, and perception were found to be highest in Khyber Pakhtunkhwa, followed by Punjab and Balochistan mostly, whereas poor knowledge, attitude, and perception were found to be highest in Azad Jammu Kashmir (Supplementary Table 4). Among various occupations/professions, veterinarians were found to have good positive knowledge, attitude, and perception toward Leishmania, while para vets were found to have poor knowledge, attitude, and perception (Supplementary Table 5). The finding of this study revealed that bachelor’s degree enrolled (4^th^ & 5^th^) students had significantly higher knowledge, attitude, and perception toward Leishmania, a good prevention practice toward Leishmania and One Health concept followed Master degree students as compared to others which might be due to more participants from Bachelor and Master degrees and having fresh knowledge Leishmania (Supplementary Table 6). Similarly, participants from government hospitals were observed with good KAP which might be due to their higher interaction with Leishmania patients (Supplementary Table 7).

## 4 Discussion

Leishmaniasis, a zoonotic vector-borne disease caused by protozoan parasites of the Leishmania genus, affects millions globally (19). Public knowledge and perception about leishmaniasis play a critical role in its prevention, particularly in endemic areas (23). An effective Leishmania control and management strategy requires a comprehensive understanding of the local population’s knowledge, attitudes, and perceptions (KAP) regarding the disease. By assessing KAP levels, targeted control programs can be more effectively designed and implemented within communities. Cutaneous leishmaniasis (CL) is highly prevalent in Pakistan and continues to expand into previously unaffected regions (24, 25). Research indicates that regions adjacent to areas endemic with Cutaneous Leishmaniasis (CL) may also face significant risk (26). The primary objective of this study is to assess the knowledge, attitudes, and practices (KAP) of individuals living in a Leishmania-endemic zone in Pakistan. The findings could play a critical role in refining strategies for Leishmania prevention and control. A comprehensive understanding of the local population’s knowledge, beliefs, and perceptions, coupled with the patterns of disease occurrence, is essential for formulating effective control and management interventions. This investigation was conducted as a cross-sectional survey targeting medical and veterinary professionals, as well as students, to evaluate their awareness and perspectives regarding leishmaniasis in Pakistan.

In our study, most of the respondents had good knowledge about Leishmania and its causative agent, which means that the disease is familiar in the community. In our study, the higher positive responses may be due to the participants of medical and veterinary professionals and students. This increased awareness may be attributed to the accessibility of information through various media platforms, particularly the Internet. Our Knowledge, Attitudes, and Practices (KAP) study indicates higher levels of understanding compared to previous research conducted in Punjab, Pakistan, where participants demonstrated insufficient knowledge about the disease and its pathogenesis (27). Similarly, a study in Colombia found that 83% of respondents were informed about leishmaniasis (28). In contrast, a study from India reported that only 38% of participants could recognize images of cutaneous leishmaniasis (CL) patients, reflecting a relatively low level of awareness and understanding of the disease (29). Our findings reveal a higher level of awareness compared to a study conducted in the highly endemic region of Waziristan, Pakistan, where only one-third of the participants were familiar with cutaneous leishmaniasis (CL), indicating overall insufficient knowledge. In contrast, our results align with a study from northeastern Ethiopia, where 76.8% of the population recognized the term Leishmania (30). The observed discrepancies may be attributed to differences in the study periods, population characteristics, data collection methods, and sample selection. Additionally, the previous research employed relatively small sample sizes, which may have affected the generalizability of the findings.

Regarding the transmission of disease, a considerable number of respondents had good knowledge about their transmission, which exceeds the Participants from endemic communities of Ghana, which were mostly unaware of their transmission (31). A similar study conducted in Saudi Arabia found that only (37.4%) of participants were aware of the transmission of leishmaniasis (32). Our respondents had better knowledge regarding the transmission of leishmaniasis than the study conducted in the southern districts of Khyber Pakhtunkhwa, where respondents were very poor, as only a few knew about the transmission of the disease. In Punjab, Pakistan, a limited understanding of leishmaniasis transmission has been highlighted by (27). This is in agreement with findings from Singh et al. in Bihar, India, where a higher level of awareness about disease transmission was observed (33). The study reported that 48% of participants recognized the possibility of human-to-human transmission, a rate exceeding the 34.4% recorded in Punjab, Pakistan (27). Enhanced community awareness regarding transmission pathways plays a crucial role in reducing the prevalence of leishmaniasis (33). The current investigation revealed that most participants exhibited a favorable perception regarding the severity of the disease, with 55.2% of the population identifying Leishmania as a fatal illness. This aligns with the studies carried out in India and Pakistan, where respondents exhibited a positive attitude of 78% (27), and 71% (33) regarding the seriousness of the disease. The findings of our study align with those from research conducted in Northwest Ethiopia, where a significant portion of participants recognized CL as a serious disease (34). Contrary to our findings, another study indicated that 61.1% of participants perceived CL as a prevalent health issue in their area (30). In a similar vein, studies conducted in Southern Ethiopia (35), and Tunisia (36) indicated that a majority of participants perceived Leishmania as a non-lethal disease. In a similar vein, merely 10% of the survey participants in Paraguay believed that leishmaniasis poses an issue (37). In the context of medical treatment, a significant proportion of study participants demonstrated confidence in their treatment decisions. Notably, 79.6% preferred antiprotozoal medications as the primary treatment choice, reflecting a solid understanding of disease management strategies among the respondents. This contrasts with a study from the Delanta district in Northeast Ethiopia, where approximately 50.5% of participants favored traditional medicine as their preferred treatment, consistent with previous Ethiopian research (34, 35), and a similar study conducted in India involving adults over 18 years (29). In our research, 72% of participants reported no direct experience with Leishmania, either personally or within their households, while the remaining 28%—mainly male participants—had observed or experienced the disease. These findings are in line with previous studies in Ethiopia, Saudi Arabia, and Pakistan, which highlight a higher incidence of cutaneous leishmaniasis (CL) among males compared to females (27, 38). This trend may be linked to the more frequent engagement of males in outdoor activities.

In our study, the proportion of participants involved in animal husbandry was lower than that observed in Dir, Khyber Pakhtunkhwa, where 79% reported having an animal shed within or adjacent to their homes (39). A cross-sectional study conducted in Dargai, located near Lower Dir District, identified domestic animals as a major source of cutaneous leishmaniasis (CL) transmission (26). There is evidence linking CL with livestock, as animal shelters create ideal breeding environments for sand flies, thereby increasing human-vector contact (40). Furthermore, CL cases have been documented in dogs living in compounds and nearby areas across various endemic regions in Pakistan (41).

In our study, 56.4% of respondents indicated that multiple initiatives had been implemented within their community to control sandfly populations. A significant portion of participants adopted preventive measures against cutaneous leishmaniasis, with 44.9% using bed nets, 27.7% relying on insecticides, and 14.7% engaging in environmental management practices, such as clearing vegetation. These findings align with those reported (34), where a substantial number of participants believed that CL could be prevented through personal hygiene measures. Similarly, a study conducted (33) in India revealed widespread awareness regarding the transmission, control, and prevention of the disease. However, in contrast to our findings, research from Dir, Khyber Pakhtunkhwa showed that only 4% of participants used bed nets, and 79% lived in unsanitary conditions (39). Recent studies indicate that a significant proportion (55%) of participants were unaware of the vectors associated with leishmaniasis and their management (27). Furthermore, only 25% recognized summer as the peak season for sand-fly bites (34, 42). Communities need to comprehend the characteristics of disease vectors and be aware of the seasonal and daily patterns of bites to effectively implement preventive measures and pursue timely treatment. Health education plays a vital role in improving the knowledge, attitudes, and practices of vulnerable populations, thereby contributing to the prevention of cutaneous leishmaniasis (CL) at both individual and community levels (43). A majority of participants acknowledged that heightened awareness could reduce the risk of CL.

The current study revealed that males had significantly higher knowledge, attitude, and perception toward Leishmania’s good prevention practices toward Leishmania and One Health Concept as compared to females. Similarly, more preventive measures were adopted by males, which is in line with (44) where males had good positive attitudes and had adopted more preventive measures than females.

Bloodborne parasitic diseases are one of the production-limiting factors in animals (45, 46). The global proliferation of zoonotic diseases, which includes infectious pathogens that are transmitted between animals and humans, poses a significant threat to public health. To effectively prevent and control these zoonotic diseases, a One Health approach is crucial (47). Additionally, further studies are important for evaluating the risks posed to humans who frequent these areas (48). This strategy emphasizes the need for collaboration across various sectors responsible for human health, animal health (both domestic and wildlife), and environmental health (47). With over 60% of human infectious diseases being zoonotic (49), it is essential to recognize the interdependence of human beings, animals, and their shared environments, which include hosts and vectors. Consequently, the implementation of a One Health strategy is vital for the effective management of leishmaniasis. This concept promotes a comprehensive, global approach that fosters collaboration across multiple disciplines and sectors in addressing all aspects of human, animal, and environmental health, highlighting their interconnected nature (50). The increased knowledge, attitude, and perception toward the One Health Approach in our study might be due to the participation of educated participants mostly belonging to health professionals. Moreover, more than half of the participants had also received formal education or training on One Health concepts, as One Health concept is mostly part of the syllabus of Health professionals.

### 4.1 Study limitations and strengths

Several limitations should be considered while evaluating the findings of this research. First, our study was limited to medical and veterinary professionals and students. Secondly, we utilized different social media platforms for data collection, but there is the possibility that some platforms were missed. Thirdly, the main author of this study is from Khyber Pakhtunkhwa, which led to a large amount of data being collected from this province, while data from other provinces was limited. This may have introduced a bias favoring Khyber Pakhtoonkhwa. Although we made efforts to gather data from other administrative units, our contacts in those areas were limited. The main strength of our study is that it provided an opportunity for individuals with more knowledge about the disease to participate, which resulted in more accurate and positive findings. These findings can serve as a valuable resource for policymakers and public health personnel in disease control and prevention efforts.

## 5 Conclusion

This cross-sectional study provides valuable insights into the knowledge, attitudes, and practices (KAP) regarding leishmaniasis among a diverse population of 5,074 participants, primarily comprising young males from rural areas of Khyber Pakhtunkhwa. The findings indicate a high level of awareness about leishmaniasis, with 96.1% of respondents familiar with the disease and a significant majority recognizing it as a zoonotic infection. Notably, younger participants demonstrated better knowledge, attitudes, and perceptions compared to older individuals, suggesting that targeted educational interventions could further enhance understanding, especially in rural settings. The study also highlights a strong connection between awareness of the One Health concept and positive attitudes toward leishmaniasis prevention, with 73.3% of participants believing that knowledge of One Health can aid in disease control. Despite the overall positive KAP scores, disparities based on gender and professional background indicate areas needing further attention, particularly among women and para-veterinary professionals. In summary, while the high levels of awareness and positive attitudes are promising, there remains an opportunity to strengthen community practices and knowledge through continued education and targeted health interventions, particularly in rural areas. Future research could focus on evaluating the effectiveness of these educational initiatives in fostering better prevention practices against leishmaniasis and improving overall public health outcomes.

## Data Availability

The original contributions presented in the study are included in the article/Supplementary material, further inquiries can be directed to the corresponding authors.
