# Supplementary material for: Knowledge, attitudes, and practices toward leishmaniasis and one health: a cross-sectional study among medical and veterinary professionals
Source: Front Vet Sci. 2025 Jan 24;11:1515370. doi: 10.3389/fvets.2024.1515370 (PMC11802810; doi:10.3389/fvets.2024.1515370)
Supplement: Supplementary file 1 [file Data_Sheet_1.pdf]

Supplementary Material

Knowledge, Attitudes, and Practices Toward Leishmaniasis and One Health: A Cross-Sectional Study Among Medical and Veterinary Professionals

Yasir Khan[1#], I-Chen Lin[2#], Sundus Khan[3], Mehtab Kanwal[4], Abdul Wajid[5], Aamir Khan[6], Fazal Noor[7], Ali Madi Almajwal[8], Chien-Chin Chen[9,10,11,12]*, Abdul Qadeer[13*],

1. Lady Reading Hospital Peshawar-Pakistan, Email: yawan4526@gmail.com
2. Division of Colorectal Surgery, Department of Surgery, Ditmanson Medical Foundation Chia-Yi Christian Hospital, Chiayi 600, Taiwan. Email: 07148@cych.org.tw
3. Kuwait Teaching Hospital Peshawar-Pakistan Email: sunduskhanpmc@gmail.com
4. Institute of Zoological Sciences, University of Peshawar-Pakistan, Email: mehtabkhan3276@gmail.com
5. Faculty of Pharmacy, Gomal University Dera Ismail Khan, Khyber Pakhtunkhwa, Email: wajidktk854@gmail.com
6. Livestock and Dairy Development Department (Extension wing), Khyber Pakhtunkhwa, Pakistan Email: aamirkhanbannuzai@gmail.com
7. Livestock and Dairy Development Department (Research wing), Khyber Pakhtunkhwa, Pakistan Email: fazalnoor555@gmail.com
8. Department of Community Health Sciences, College of Applied Medical Sciences, King Saud University, P.O. Box 10219 Riyadh 11433, Saudi Arabia, Email: aalmajwal@ksu.edu.sa
9. Department of Pathology, Ditmanson Medical Foundation Chia-Yi Christian Hospital, Chiayi 600, Taiwan; Email: hlmarkc@gmail.com
10. Department of Cosmetic Science, Chia Nan University of Pharmacy and Science, Tainan 717, Taiwan.
11. Doctoral Program in Translational Medicine, National Chung Hsing University, Taichung 402, Taiwan.
12. Department of Biotechnology and Bioindustry Sciences, College of Bioscience and Biotechnology, National Cheng Kung University, Tainan, 701, Taiwan.

13. Department of Cell Biology, School of Life Sciences, Central South University, Changsha, The People's Republic of China. Email: qadeerktk848@csu.edu.cn

14.
15.
16.
17.
18.

S. Table 1: Association of knowledge, attitude, and perception with the demographic variable age of the participant

Question	Variable	Unique Variable	+Ve Knowledge %	-Ve Knowledge %	Chi-square	Significance level (p-value)
**Have you heard about Leishmania?**	Age	20-35	80.6	2.0	3.478	0.324
		36-50	13.6	0.4		
		51-65	1.3	0.1		
		Undisclosed	1.9	0.0		
**What is the causative agent of Leishmania?**	Age	20-35	71.1	11.5	114.301	0.000
		36-50	12.0	2.0		
		51-65	1.2	0.2		
		Undisclosed	1.7	0.3		
**Is Leishmaniasis an infectious disease?**	Age	20-35	33.2	42.9	131.424	0.00
		36-50	8.3	4.5		
		51-65	0.3	1.1		
		Undisclosed	0.8	1.0		
**Is Leishmania contagious?**	Age	20-35	33.2	43.0	127.185	0.000
		36-50	8.3	4.5		
		51-65	0.3	1.1		

		Undisclosed	0.8	1.0		
**Do you hear about mortality from leishmania?**	Age	20-35	45.5	30.5	11.242	0.000
		36-50	7.7	4.9		
		51-65	0.8	0.6		
		Undisclosed	1.1	0.8		
**Is leishmania transmitted from human to human?**	Age	20-35	39.0	33.2	19.709	0.003
		36-50	7.3	4.8		
		51-65	0.8	0.6		
		Undisclosed	0.9	0.7		
**Is leishmania transmitted from animal to animal?**	Age	20-35	52.9	20.8	23.185	0.001
		36-50	9.8	3.3		
		51-65	1.1	0.3		
		Undisclosed	1.3	0.5		
**Is leishmania transmitted from animal to human?**	Age	20-35	59.2	16.9	9.503	0.147
		36-50	10.0	2.7		
		51-65	0.9	0.3		
		Undisclosed	1.4	0.4		
**Is leishmania transmitted from human to animal?**	Age	20-35	31.5	33.5	12.369	0.054
		36-50	6.1	4.9		
		51-65	0.6	0.6		

		Undisclosed	0.6	0.9		
**Does leishmania transmit from pregnant women to their offspring?**	Age	20-35	41.4	26.9	103.811	0.000
		36-50	8.1	3.0		
		51-65	0.3	1.1		
		Undisclosed	1.0	0.6		
**Does leishmania transmit from infected animals meat or milk?**	Age	20-35	24.7	43.3	124.870	0.00
		36-50	5.1	5.3		
		51-65	0.0	1.4		
		Undisclosed	0.6	0.9		
**Have you ever heard about leishmania treatment?**	Age	20-35	71.9	6.8	66.366	0.000
		36-50	13.1	0.2		
		51-65	1.1	0.3		
		Undisclosed	1.6	0.2		
**If 1, what treatment is done for Leishmania infection?**	Age	20-35	64.9	17.7	78.165	0.000
		36-50	11.8	2.3		
		51-65	1.4	0.0		
		Undisclosed	1.6	0.4		
**Have you heard of the**	Age	20-35	74.4	8.2	2.450	0484

**term "One Health"?**		36-50	12.7	1.4		
		51-65	1.3	0.1		
		Undisclosed	1.8	0.1		
**Have you received any formal education or training on One Health concepts?**	Age	20-35	42.4	40.2	68.670	0.000
		36-50	9.4	4.6		
		51-65	0.6	0.9		
		Undisclosed	1.2	0.8		
**Do you Know Zoonotic?**	Age	20-35	79.0	3.6	3.406	0.333
		36-50	13.3	0.7		
		51-65	1.4	0.0		
		Undisclosed	1.9	0.1		
**Is leishmania a Zoonotic disease?**	Age	20-35	73.5	9.1	4.826	0.185
		36-50	12.3	1.7		
		51-65	1.3	0.1		
		Undisclosed	1.8	0.1		
**Have you or anyone in your household ever been diagnosed with leishmaniasis?**	Age	20-35	22.0	60.6	100.478	0.000
		36-50	5.9	8.1		
		51-65	0.0	1.4		

		Undisclosed	0.4	1.6		
**Are there measures taken in your community to control sandfly populations?**	Age	20-35	46.4	36.2	42.828	0.000
		36-50	8.7	5.3		
		51-65	0.3	1.1		
		Undisclosed	1.0	1.0		
**Do you use bed nets daily?**	Age	20-35	37.2	45.4	66.418	0.000
		36-50	7.7	6.3		
		51-65	0.1	1.3		
		Undisclosed	0.7	1.2		
**Do you think having knowledge about One Health can help in preventing leishmaniasis?**	Age	20-35	61.8	4.9	4.454	0.615
		36-50	10.2	1.0		
		51-65	1.1	0.0		
		Undisclosed	1.5	0.1		
**Have you implemented any preventive measures in your home or community based on your**	Age	20-35	63.0	19.6	15010	0.002
		36-50	11.1	3.0		

**knowledge of One Health?**		51-65	0.8	0.6		
		Undisclosed	1.6	0.4		
**Are there any community initiatives or programs promoting One Health concepts in your area?**	Age	20-35	38.8	49.8	106.263	0.000
		36-50	8.5	5.6		
		51-65	0.6	0.9		
		Undisclosed	0.7	1.2		
**If 1, are you involved in any of these initiatives?**	Age	20-35	26.1	56.5	121.573	0.000
		36-50	7.3	6.7		
		51-50	0.4	1.1		
		Undisclosed	0.5	1.4		

+Ve = Positive –ive=Negative

19. S. Table: 02 Association of knowledge, attitude, and perception with the demographic variable Gender of participant

**Question**	**Variable**	**Unique Variable**	**+Ve Knowledge %**	**-Ve Knowledge**	**Chi-square**	**Significance level (p-value)**
**Have you heard about Leishmania?**	Gender	Female	17.1	0.6	1.981	0.349
		Male	80.4	1.9		
**What is the causative agent of Leishmania?**	Gender	Female	15.3	2.4	21.608	0.001
		Male	70.7	11.6		

**Is Leishmaniasis an infectious disease?**	Gender	Female	9.4	6.2	93.815	0.000
		Male	33.2	43.3		
**Is Leishmania contagious?**	Gender	Female	9.4	6.2	94.819	0.000
		Male	33.3	43.4		
**Do you hear about mortality from leishmania?**	Gender	Female	9.9	5.3	64.474	0.000
		Male	45.3	31.5		
**Is leishmania transmitted from human to human?**	Gender	Female	9.4	5.8	18.977	0.000
		Male	38.6	32.5		
**Is leishmania transmitted from animal to animal?**	Gender	Female	12.4	3.9	12.965	0.002
		Male	52.7	20.9		
**Is leishmania transmitted from animal to human?**	Gender	Female	14.9	2.4	101.619	0.000
		Male	56.9	17.9		
**Is leishmania transmitted from human to animal?**	Gender	Female	6.0	7.1	16.322	0.000
		Male	32.8	32.8		
**Does leishmania transmit from pregnant women to their offspring?**	Gender	Female	11.8	4.3	115.433	0.000
		Male	39.1	27.4		
**Does leishmania transmit from infected animals meat or milk?**	Gender	Female	7.0	7.1	55.130	0.000
		Male	23.4	43.8		

**Have you ever heard about leishmania treatment?**	Gender	Female	16.3	1.3	50.111	0.000
		Male	71.4	6.2		
**If 1, what treatment is done for Leishmania infection?**	Gender	Female	13.5	4.2	81.158	0.000
		Male	66.2	16.1		
**Have you heard of the term "One Health"?**	Gender	Female	15.9	1.8	0.065	0.419
		Male	74.3	8.0		
**Have you received any formal education or training on One Health concepts?**	Gender	Female	10.3	7.4	10.176	0.001
		Male	43.2	39.1		
**Do you Know Zoonotic?**	Gender	Female	17.1	0.6	3.127	0.043
		Male	78.5	3.9		
**Is leishmania a Zoonotic disease?**	Gender	Female	15.9	1.7	1.537	0.118
		Male	73.1	9.3		
**Have you or anyone in your household ever been diagnosed with leishmaniasis?**	Gender	Female	4.9	12.8	0.337	0296
		Male	23.5	58.8		
**Are there measures taken in your community to control sandfly populations?**	Gender	Female	11.7	6.8	43.279	0.000
		Male	44.7	37.6		
**Do you use bed nets daily?**	Gender	Female	9.0	8.6	12.998	0.000
		Male	36.7	45.6		

**Do you think having knowledge about One Health can help in preventing leishmaniasis?**	Gender	Female	13.3	1.6	24.668	0.000
		Male	61.3	4.4		
**Have you implemented any preventive measures in your home or community based on your knowledge of One Health?**	Gender	Female	14.7	2.9	28.368	0.000
		Male	61.8	20.5		
**Are there any community initiatives or programs promoting One Health concepts in your area?**	Gender	Female	9.6	8.1	62.007	0.000
		Male	33.0	49.4		
**If 1, are you involved in any of these initiatives?**	Gender	Female	7.5	10.1	34.849	0.000
		Male	26.7	55.6		

20.
21. S. Table 3: Association of knowledge, attitude, and perception with the demographic variable residence of the participant

**Question**	**Variable**	**Unique Variable**	**+Ve Knowledge %**	**-Ve Knowledge**	**Chi-square**	**Significance level (p-value)**
**Have you heard about Leishmania?**	Residence	Urban	48.3	0.9	8.977	0.003
		Rural	49.2	1.6		
**What is the causative agent of Leishmania?**	Residence	Urban	42.4	6.8	31.765	0.000
		Rural	43.6	7.2		

**Is Leishmaniasis an infectious disease?**	Residence	Urban	21.4	23.3	19.641	0.000
		Rural	21.2	26.3		
**Is Leishmania contagious?**	Residence	Urban	21.4	23.2	21.658	0.000
		Rural	21.3	26.4		
**Do you hear about mortality from leishmania?**	Residence	Urban	27.3	17.7	3.046	0.218
		Rural	27.9	19.1		
**Is leishmania transmitted from human to human?**	Residence	Urban	21.9	19.1	79.976	0.000
		Rural	26.1	20.3		
**Is leishmania transmitted from animal to animal?**	Residence	Urban	33.3	10.4	41.471	0.000
		Rural	31.8	14.5		
**Is leishmania transmitted from animal to human?**	Residence	Urban	34.6	10.7	5.951	0.051
		Rural	37.2	9.7		
**Is leishmania transmitted from human to animal?**	Residence	Urban	18.1	17.4	128.567	0.000
		Rural	20.8	22.4		
**Does leishmania transmit from pregnant women to their offspring?**	Residence	Urban	25.5	13.4	62.927	0.000
		Rural	25.3	18.2		
**Does leishmania transmit from infected animals meat or milk?**	Residence	Urban	16.2	21.5	121.473	0.000
		Rural	14.2	29.4		

**Have you ever heard about leishmania treatment?**	Residence	Urban	43.2	3.4	5.387	0.068
		Rural	44.5	4.1		
**If 1, what treatment is done for Leishmania infection?**	Residence	Urban	39.3	9.9	16.386	0.001
		Rural	40.3	10.5		
**Have you heard of the term "One Health"?**	Residence	Urban	44.1	5.1	1.959	0.162
		Rural	46.1	4.7		
**Have you received any formal education or training on One Health concepts?**	Residence	Urban	27.5	21.7	10.439	0.001
		Rural	26.1	24.7		
**Do you Know Zoonotic?**	Residence	Urban	48.0	1.2	46.810	0.000
		Rural	47.5	3.3		
**Is leishmania a Zoonotic disease?**	Residence	Urban	43.7	5.5	0.225	0.635
		Rural	45.3	5.5		
**Have you or anyone in your household ever been diagnosed with leishmaniasis?**	Residence	Urban	11.3	38.0	72.248	0.000
		Rural	17.1	33.7		
**Are there measures taken in your community to control sandfly populations?**	Residence	Urban	28.6	20.8	5.656	0.017
		Rural	27.3	23.0		
	Residence	Urban	24.5	24.8	30.730	0.000

**Do you use bed nets daily?**		Rural	21.3	29.5		
**Do you think having knowledge about One Health can help in preventing leishmaniasis?**	Residence	Urban	35.8	1.5	140.170	0.000
		Rural	38.8	4.5		
**Have you implemented any preventive measures in your home or community based on your knowledge of One Health?**	Residence	Urban	38.0	11.3	0.903	0.342
		Rural	38.6	12.2		
**Are there any community initiatives or programs promoting One Health concepts in your area?**	Residence	Urban	23.4	25.9	48.876	0.000
		Rural	19.2	31.6		
**If 1, are you involved in any of these initiatives?**	Residence	Urban	17.4	31.8	2.816	0.093
		Rural	16.8	33.9		

22.
23. S. Table 4: Association of knowledge, attitude, and perception with the demographic variable administrative Unit of participant

**Question**	**Variable**	**Unique Variable**	**+Ve Knowledge %**	**-Ve Knowledge**	**Chi-square**	**Significance level (p-value)**
**Have you heard about Leishmania?**	Administrative Unit	Azad Jammu Kashmir	2.5	0.0	36.562	0.000
		Balochistan	12.7	0.0		
		Gilgit Baltistan	2.2	0.0		

		Islamabad Capital Territory	4.4	0.0		
		Khyber Pakhtoonkhwa	55.6	1.9		
		Punjab	13.5	0.2		
		Sindh	6.6	0.3		
**What is the causative agent of Leishmania?**	Administrative Unit	Azad Jammu Kashmir	2.4	0.1	445.982	0.000
		Balochistan	11.0	1.8		
		Gilgit Baltistan	2.2	0.0		
		Islamabad Capital Territory	4.1	0.3		
		Khyber Pakhtoonkhwa	49.5	7.9		
		Punjab	11.5	2.2		
		Sindh	5.3	1.7		
**Is Leishmaniasis an infectious disease?**	Administrative Unit	Azad Jammu Kashmir	1.6	0.9	733.259	0.000
		Balochistan	10.2	2.3		
		Gilgit Baltistan	0.3	1.9		
		Islamabad Capital Territory	2.8	0.9		
		Khyber Pakhtoonkhwa	19.1	33.9		

		Punjab	5.5	7.3		
		Sindh	3.1	2.4		
**Is Leishmania contagious?**	Administrative Unit	Azad Jammu Kashmir	1.6	0.9	735.556	0.000
		Balochistan	10.2	2.3		
		Gilgit Baltistan	0.3	1.9		
		Islamabad Capital Territory	2.8	0.9		
		Khyber Pakhtoonkhwa	19.1	33.9		
		Punjab	5.5	7.3		
		Sindh	3.1	2.4		
**Do you hear about mortality from leishmania?**	Administrative Unit	Azad Jammu Kashmir	0.9	1.6	415.311	0.000
		Balochistan	9.5	1.6		
		Gilgit Baltistan	0.6	1.6		
		Islamabad Capital Territory	3.2	0.7		
		Khyber Pakhtoonkhwa	31.2	22.6		
		Punjab	5.7	6.9		
		Sindh	4.1	1.9		
**Is leishmania transmitted**	Administrative Unit	Azad Jammu Kashmir	1.6	0.9	499.501	0.000

**from human to human?**		Balochistan	4.7	4.9		
		Gilgit Baltistan	0.3	1.9		
		Islamabad Capital Territory	2.3	0.3		
		Khyber Pakhtoonkhwa	27.2	24.9		
		Punjab	7.6	4.5		
		Sindh	4.3	1.9		
**Is leishmania transmitted from animal to animal?**	Administrative Unit	Azad Jammu Kashmir	2.0	0.5	302.371	0.000
		Balochistan	11.0	1.1		
		Gilgit Baltistan	1.3	0.9		
		Islamabad Capital Territory	3.5	0.0		
		Khyber Pakhtoonkhwa	33.9	17.2		
		Punjab	9.1	3.3		
		Sindh	4.3	1.9		
	Administrative Unit	Azad Jammu Kashmir	2.0	0.1	240.489	0.000
		Balochistan	11.0	0.9		
		Gilgit Baltistan	2.2	0.0		

**Is leishmania transmitted from animal to human?**		Islamabad Capital Territory	3.8	0.3		
		Khyber Pakhtoonkhwa	38.3	14.7		
		Punjab	9.3	2.9		
		Sindh	5.3	1.4		
**Is leishmania transmitted from human to animal?**	Administrative Unit	Azad Jammu Kashmir	0.9	0.9	410.198	0.000
		Balochistan	5.1	3.5		
		Gilgit Baltistan	0.3	1.9		
		Islamabad Capital Territory	1.6	0.3		
		Khyber Pakhtoonkhwa	21.3	24.9		
		Punjab	6.2	5.7		
		Sindh	3.4	2.6		
**Does leishmania transmit from pregnant women to their offspring?**	Administrative Unit	Azad Jammu Kashmir	1	1.5	532.995	0.000
		Balochistan	8.8	1.3		
		Gilgit Baltistan	2.2	0.0		
		Islamabad Capital Territory	3.4	0.3		
		Khyber Pakhtoonkhwa	23.3	22.7		

		Punjab	7.6	4.1		
		Sindh	4.5	1.7		
**Does leishmania transmit from infected animals meat or milk?**	Administrative Unit	Azad Jammu Kashmir	0.9	1.5	502.739	0.000
		Balochistan	5.5	3.5		
		Gilgit Baltistan	0.3	1.9		
		Islamabad Capital Territory	1.6	1.0		
		Khyber Pakhtoonkhwa	13.3	34.5		
		Punjab	5.7	6.0		
		Sindh	3.1	2.5		
**Have you ever heard about leishmania treatment?**	Administrative Unit	Azad Jammu Kashmir	2.2	0.4	217.394	0.000
		Balochistan	11.5	0.1		
		Gilgit Baltistan	2.2	0.0		
		Islamabad Capital Territory	4.4	0.0		
		Khyber Pakhtoonkhwa	49.8	5.0		
		Punjab	11.0	2.0		
		Sindh	6.6	0.0		
**If 1, what treatment is done for**	Administrative Unit	Azad Jammu Kashmir	2.4	0.1	273.563	0.000

**Leishmania infection?**		Balochistan	10.0	2.8		
		Gilgit Baltistan	2.2	0.0		
		Islamabad Capital Territory	3.4	1.0		
		Khyber Pakhtoonkhwa	45.9	11.5		
		Punjab	11.1	2.6		
		Sindh	4.7	0.0		
**Have you ever heard of the term "One Health"?**	Administrative Unit	Azad Jammu Kashmir	2.5	0.0	167.914	0.000
		Balochistan	12.3	0.4		
		Gilgit Baltistan	2.2	0.0		
		Islamabad Capital Territory	4.1	0.3		
		Khyber Pakhtoonkhwa	49.2	8.3		
		Punjab	13.2	0.5		
		Sindh	6.6	0.3		
**Have you received any formal education or training on One Health concepts?**	Administrative Unit	Azad Jammu Kashmir	1.6	0.9	623.550	0.000
		Balochistan	11.0	1.8		
		Gilgit Baltistan	0.7	1.6		
		Islamabad Capital Territory	3.5	0.9		

		Khyber Pakhtoonkhwa	23.5	34.0		
		Punjab	8.3	5.4		
		Sindh	5.0	1.9		
**Do you Know Zoonotic?**	Administrative Unit	Azad Jammu Kashmir	2.5	0.0	69.615	0.000
		Balochistan	12.2	0.6		
		Gilgit Baltistan	2.2	0.0		
		Islamabad Capital Territory	3.8	0.7		
		Khyber Pakhtoonkhwa	55.2	2.3		
		Punjab	13.0	0.7		
		Sindh	6.6	0.3		
**Is leishmania a Zoonotic disease?**	Administrative Unit	Azad Jammu Kashmir	2.5	0.0	68.567	0.000
		Balochistan	11.8	0.9		
		Gilgit Baltistan	2.2	0.0		
		Islamabad Capital Territory	3.5	0.9		
		Khyber Pakhtoonkhwa	50.4	7.1		
		Punjab	12.3	1.4		
		Sindh	6.4	0.6		

**Have you or anyone in your household ever been diagnosed with leishmaniasis?**	Administrative Unit	Azad Jammu Kashmir	0.5	2.0	177.7092	0.000
		Balochistan	3.8	9.0		
		Gilgit Baltistan	0.0	2.2		
		Islamabad Capital Territory	2.3	2.1		
		Khyber Pakhtoonkhwa	15.8	41.6		
		Punjab	2.9	10.9		
		Sindh	3.1	3.8		
**Are there measures taken in your community to control sandfly population?**	Administrative Unit	Azad Jammu Kashmir	1.7	0.9	576.201	0.000
		Balochistan	11.3	1.5		
		Gilgit Baltistan	1.0	1.2		
		Islamabad Capital Territory	4.4	0.0		
		Khyber Pakhtoonkhwa	27.7	29.8		
		Punjab	6.1	7.6		
		Sindh	4.3	2.6		
**Do you use bed nets daily?**	Administrative Unit	Azad Jammu Kashmir	0.2	2.3	1003.141	0.000
		Balochistan	11.3	1.5		
		Gilgit Baltistan	0.7	1.6		

		Islamabad Capital Territory	4.1	0.3		
		Khyber Pakhtoonkhwa	20.6	36.8		
		Punjab	4.3	9.5		
		Sindh	4.6	2.3		
**Do you think having knowledge about One Health can help in preventing leishmaniasis?**	Administrative Unit	Azad Jammu Kashmir	1.7	0.4	106.775	0.000
		Balochistan	8.9	0.7		
		Gilgit Baltistan	2.2	0.00		
		Islamabad Capital Territory	2.9	0.3		
		Khyber Pakhtoonkhwa	43.1	3.8		
		Punjab	10.4	0.7		
		Sindh	5.4	0.0		
**Have you implemented any preventive measures in your home or community based on your knowledge of One Health?**	Administrative Unit	Azad Jammu Kashmir	2.0	0.6	212.868	0.000
		Balochistan	11.8	1.0		
		Gilgit Baltistan	1.3	0.9		
		Islamabad Capital Territory	4.4	0.0		
		Khyber Pakhtoonkhwa	42.1	15.3		

		Punjab	9.7	4.1		
		Sindh	5.3	1.6		
**Are there any community initiatives or programs promoting One Health concepts in your area?**	Administrative Unit	Azad Jammu Kashmir	0.8	1.7	782.405	0.000
		Balochistan	10.3	2.5		
		Gilgit Baltistan	0.9	1.3		
		Islamabad Capital Territory	4.1	0.3		
		Khyber Pakhtoonkhwa	18.2	39.2		
		Punjab	4.7	9.0		
		Sindh	3.4	3.5		
**If 1, are you involved in any of these initiatives?**	Administrative Unit	Azad Jammu Kashmir	0.5	2.0	334.209	0.000
		Balochistan	6.5	6.2		
		Gilgit Baltistan	1.3	0.9		
		Islamabad Capital Territory	3.2	1.2		
		Khyber Pakhtoonkhwa	16.0	41.4		
		Punjab	3.8	9.9		
		Sindh	2.9	4.0		

24.

25. S. Table 5: Association of knowledge, attitude, and perception with the demographic variable Occupation/profession of participant

Question	Variable	Unique Variable	+Ve Knowledge %	-Ve Knowledge	Chi-square	Significance level (p-value)
**Have you heard about Leishmania?**	Occupation / Profession	Student (Medical/ Veterinary)	26.1	1.3	130.948	0.000
		Veterinary Doctor	42.1	0.1		
		Medical Doctor	14.9	0.3		
		Paramedic Staff	7.8	0.7		
		Paravet staff	6.6	0.1		
**What is the causative agent of Leishmania?**	Occupation / Profession	Student (Medical/ Veterinary)	22.9	4.5	588.199	0.000
		Veterinary Doctor	38.9	3.2		
		Medical Doctor	13.6	1.6		
		Paramedic Staff	5.7	2.8		
		Paravet staff	4.9	1.9		
**Is Leishmaniasis an infectious disease?**	Occupation / Profession	Student (Medical/ Veterinary)	9.2	14.7	446.948	0.000
		Veterinary Doctor	15.0	25.1		
		Medical Doctor	8.4	6.0		

		Paramedic Staff	6.0	2.0		
		Paravet staff	4.1	1.7		
**Is Leishmania contagious?**	Occupation / Profession	Student (Medical/ Veterinary)	9.2	14.8	443.133	0.000
		Veterinary Doctor	15.1	25.1		
		Medical Doctor	8.4	6.0		
		Paramedic Staff	6.0	2.0		
		Paravet staff	4.1	1.8		
**Do you hear about mortality from leishmania?**	Occupation / Profession	Student (Medical/ Veterinary)	14.7	10.2	360.772	0.000
		Veterinary Doctor	19.9	19.9		
		Medical Doctor	9.9	4.3		
		Paramedic Staff	5.3	2.3		
		Paravet staff	5.4	0.1		
**Is leishmania transmitted from human to human?**	Occupation / Profession	Student (Medical/ Veterinary)	14.1	10.8	180.599	0.000
		Veterinary Doctor	19.9	18.0		
		Medical Doctor	6.0	6.4		
		Paramedic Staff	4.7	2.8		

		Paravet staff	3.4	1.4		
**Is leishmania transmitted from animal to animal?**	Occupation / Profession	Student (Medical/ Veterinary)	19.4	4.9	209.715	0.000
		Veterinary Doctor	26.0	12.4		
		Medical Doctor	7.6	5.3		
		Paramedic Staff	6.5	1.5		
		Paravet staff	5.6	0.7		
**Is leishmania transmitted from animal to human?**	Occupation / Profession	Student (Medical/ Veterinary)	19.6	5.3	64.340	0.000
		Veterinary Doctor	30.1	9.7		
		Medical Doctor	11.7	2.4		
		Paramedic Staff	5.9	1.7		
		Paravet staff	4.5	1.3		
**Is leishmania transmitted from human to animal?**	Occupation / Profession	Student (Medical/ Veterinary)	13.1	8.8	289.275	0.000
		Veterinary Doctor	14.8	20.8		
		Medical Doctor	4.0	5.8		
		Paramedic Staff	3.8	2.7		
		Paravet staff	3.2	1.7		

**Does leishmania transmit from pregnant women to their offspring?**	Occupation / Profession	Student (Medical/ Veterinary)	14.9	7.8	194.906	0.000
		Veterinary Doctor	18.5	15.2		
		Medical Doctor	9.3	5.0		
		Paramedic Staff	5.1	2.0		
		Paravet staff	2.9	1.7		
**Does leishmania transmit from infected animals meat or milk?**	Occupation / Profession	Student (Medical/ Veterinary)	11.4	12.3	654.065	0.000
		Veterinary Doctor	9.0	27.3		
		Medical Doctor	3.1	7.8		
		Paramedic Staff	4.4	2.4		
		Paravet staff	2.5	1.1		
**Have you ever heard about leishmania treatment?**	Occupation / Profession	Student (Medical/ Veterinary)	22.3	3.5	290.737	0.000
		Veterinary Doctor	39.0	1.5		
		Medical Doctor	14.2	1.0		
		Paramedic Staff	7.6	0.4		
		Paravet staff	4.6	1.1		

**If 1, what treatment is done for Leishmania infection?**	Occupation / Profession	Student (Medical/ Veterinary)	21.5	5.9	466.935	0.000
		Veterinary Doctor	36.4	5.7		
		Medical Doctor	12.6	2.6		
		Paramedic Staff	4.6	3.9		
		Paravet staff	4.6	2.2		
**Have you ever heard of the term “One Health”?**	Occupation / Profession	Student (Medical/ Veterinary)	25.1	2.3	191.716	0.000
		Veterinary Doctor	39.2	3.0		
		Medical Doctor	11.6	3.5		
		Paramedic Staff	8.0	0.5		
		Paravet staff	6.2	0.5		
**Have you received any formal education or training on One Health concepts?**	Occupation / Profession	Student (Medical/ Veterinary)	15.1	12.3	265.901	0.000
		Veterinary Doctor	18.1	24.1		
		Medical Doctor	8.9	6.3		
		Paramedic Staff	6.8	1.7		
		Paravet staff	4.7	2.0		

**Do you Know Zoonotic?**	Occupation / Profession	Student (Medical/ Veterinary)	25.7	1.7	305.735	0.000
		Veterinary Doctor	41.9	0.2		
		Medical Doctor	14.9	0.3		
		Paramedic Staff	7.4	1.0		
		Paravet staff	5.6	1.2		
**Is leishmania a Zoonotic disease?**	Occupation / Profession	Student (Medical/ Veterinary)	25.9	1.5	122.989	0.000
		Veterinary Doctor	37.8	4.4		
		Medical Doctor	13.2	2.0		
		Paramedic Staff	6.9	1.6		
		Paravet staff	5.2	1.6		
**Have you or anyone in your household ever been diagnosed with leishmaniasis?**	Occupation / Profession	Student (Medical/ Veterinary)	6.2	21.2	475.155	0.000
		Veterinary Doctor	10.1	32.0		
		Medical Doctor	3.1	12.1		
		Paramedic Staff	5.9	2.6		
		Paravet staff	3.1	3.7		

**Are there measures taken in your community to control sandfly population?**	Occupation / Profession	Student (Medical/ Veterinary)	14.1	13.3	335.501	0.000
		Veterinary Doctor	19.9	22.3		
		Medical Doctor	9.8	5.4		
		Paramedic Staff	7.4	1.1		
		Paravet staff	5.2	1.6		
**Do you use bed nets daily?**	Occupation / Profession	Student (Medical/ Veterinary)	10.9	16.5	293.297	0.000
		Veterinary Doctor	16.4	25.7		
		Medical Doctor	7.3	7.9		
		Paramedic Staff	6.2	2.2		
		Paravet staff	4.9	1.9		
**Do you think having knowledge about One Health can help in preventing leishmaniasis?**	Occupation / Profession	Student (Medical/ Veterinary)	20.6	1.8	120.710	0.000
		Veterinary Doctor	33.3	2.5		
		Medical Doctor	9.5	0.8		
		Paramedic Staff	6.4	0.3		
		Paravet staff	4.8	0.6		

**Have you implemented any preventive measures in your home or community based on your knowledge of One Health?**	Occupation / Profession	Student (Medical/ Veterinary)	20.9	6.5	138.548	0.000
		Veterinary Doctor	30.3	11.8		
		Medical Doctor	12.0	3..2		
		Paramedic Staff	8.3	0.2		
		Paravet staff	5.0	1.8		
**Are there any community initiatives or programs promoting One Health concepts in your area?**	Occupation / Profession	Student (Medical/ Veterinary)	10.3	17.1	311.384	0.000
		Veterinary Doctor	14.0	28.1		
		Medical Doctor	8.7	6.5		
		Paramedic Staff	6.0	2.5		
		Paravet staff	3.6	3.2		
**If 1, are you involved in any of these initiatives?**	Occupation / Profession	Student (Medical/ Veterinary)	7.4	20.0	332.620	0.000
		Veterinary Doctor	11.8	30.3		
		Medical Doctor	5.9	2.6		
		Paramedic Staff	5.9	2.6		
		Paravet staff	3.2	3.6		

26.
27. S. Table 6: Association of knowledge, attitude, and perception with the demographic variable education of participant

Question	Variable	Unique Variable	+Ve Knowledge %	-Ve Knowledge	Chi-square	Significance level (p-value)
**Have you heard about Leishmania?**	Education	Bachelor Enrolled (4th / 5th year of degree)	26.7	1.2	39.956	0.000
		Doctorate	9.3	0.1		
		Master	25.6	0.5		
		Diploma / Intermediate	14.1	0.5		
		Bachelor	18.2	0.2		
		Post Doctorate	3.7	0.0		
**What is the causative agent of Leishmania?**	Education	Bachelor Enrolled (4th / 5th year of degree)	23.8	4.1	454.644	0.000
		Doctorate	9.2	0.1		
		Master	23.3	2.8		
		Diploma / Intermediate	10.0	4.6		
		Bachelor	16.0	2.4		
		Post Doctorate	3.6	0.00		
**Is Leishmaniasis an infectious disease?**	Education	Bachelor Enrolled (4th / 5th year of degree)	9.7	14.9	415.401	0.000
		Doctorate	4.5	4.7		
		Master	8.7	15.5		

		Diploma / Intermediate	95	3.7		
		Bachelor	9.6	8.3		
		Post Doctorate	0.8	2.6		
**Is Leishmania contagious?**	Education	Bachelor Enrolled (4th / 5th year of degree)	9.7	14.7	417.720	0.000
		Doctorate	4.6	4.7		
		Master	8.7	15.5		
		Diploma / Intermediate	9.5	3.7		
		Bachelor	9.6	8.3		
		Post Doctorate	0.8	2.6		
**Do you hear about mortality from leishmania?**	Education	Bachelor Enrolled (4th / 5th year of degree)	15.4	10.1	208.525	0.000
		Doctorate	5.1	4.1		
		Master	14.1	10.3		
		Diploma / Intermediate	9.8	2.6		
		Bachelor	9.2	8.0		
		Post Doctorate	1.5	1.6		
**Is leishmania transmitted from human to human?**	Education	Bachelor Enrolled (4th / 5th year of degree)	13.6	11.4	119.931	0.000

		Doctorate	4.5	3.8		
		Master	13.2	9.6		
		Diploma / Intermediate	7.4	4.3		
		Bachelor	8.4	7.8		
		Post Doctorate	0.9	2.3		
**Is leishmania transmitted from animal to animal?**	Education	Bachelor Enrolled (4th / 5th year of degree)	19.2	5.5	232.441	0.000
		Doctorate	5.8	2.6		
		Master	15.8	7.7		
		Diploma / Intermediate	11.4	2.2		
		Bachelor	12.0	4.6		
		Post Doctorate	1.3	2.2		
**Is leishmania transmitted from animal to human?**	Education	Bachelor Enrolled (4th / 5th year of degree)	20.6	4.7	139.157	0.000
		Doctorate	6.7	2.0		
		Master	18.5	6.2		
		Diploma / Intermediate	9.5	3.3		
		Bachelor	14.4	2.7		
		Post Doctorate	2.0	1.5		

**Is leishmania transmitted from human to animal?**	Education	Bachelor Enrolled (4th / 5th year of degree)	12.3	8.8	237.957	0.00
		Doctorate	3.5	4.6		
		Master	11.5	10.2		
		Diploma / Intermediate	6.2	4.7		
		Bachelor	4.5	9.1		
		Post Doctorate	0.7	2.4		
**Does leishmania transmit from pregnant women to their offspring?**	Education	Bachelor Enrolled (4th / 5th year of degree)	15.6	7.9	87.720	0.000
		Doctorate	3.7	4.4		
		Master	12.7	9.0		
		Diploma / Intermediate	7.7	3.7		
		Bachelor	9.3	5.6		
		Post Doctorate	1.9	1.2		
**Does leishmania transmit from infected animals meat or milk?**	Education	Bachelor Enrolled (4th / 5th year of degree)	11.0	12.5	415.979	0.000
		Doctorate	1.6	6.5		
		Master	5.7	15.4		
		Diploma / Intermediate	6.4	3.7		

		Bachelor	5.1	10.1		
		Post Doctorate	0.6	2.7		
**Have you ever heard about leishmania treatment?**	Education	Bachelor Enrolled (4th / 5th year of degree)	23.3	3.1	163.529	0.000
		Doctorate	8.8	0.4		
		Master	23.9	1.3		
		Diploma / Intermediate	11.6	1.4		
		Bachelor	16.7	1.3		
		Post Doctorate	3.4	0.00		
**If 1, what treatment is done for Leishmania infection?**	Education	Bachelor Enrolled (4th / 5th year of degree)	22.6	5.3	434.243	0.000
		Doctorate	8.6	0.7		
		Master	20.7	5.4		
		Diploma / Intermediate	9.0	5.4		
		Bachelor	15.4	3.0		
		Post Doctorate	3.3	0.4		
**Have you ever heard of the term “One Health”?**	Education	Bachelor Enrolled (4th / 5th year of degree)	25.5	2.4	34.858	0.000
		Doctorate	8.6	0.8		

		Master	23.1	3.1		
		Diploma / Intermediate	13.7	0.9		
		Bachelor	16.3	2.1		
		Post Doctorate	3.1	0.6		
**Have you received any formal education or training on One Health concepts?**	Education	Bachelor Enrolled (4th / 5th year of degree)	15.1	12.8	188.297	0.000
		Doctorate	5.3	4.1		
		Master	11.6	14.5		
		Diploma / Intermediate	10.8	3.7		
		Bachelor	9.2	9.2		
		Post Doctorate	1.6	2.1		
**Do you Know Zoonotic?**	Education	Bachelor Enrolled (4th / 5th year of degree)	26.5	1.3	265.722	0.000
		Doctorate	9.3	0.0		
		Master	25.9	0.3		
		Diploma / Intermediate	12.4	2.2		
		Bachelor	17.8	0.6		
		Post Doctorate	3.6	0.0		
		Bachelor Enrolled (4th	26.4	1.5	146.248	0.000

**Is leishmania a Zoonotic disease?**	Education	/ 5th year of degree)				
		Doctorate	8.4	0.9		
		Master	22.8	3.4		
		Diploma / Intermediate	11.4	3.2		
		Bachelor	16.6	1.8		
		Post Doctorate	3.4	0.3		
**Have you or anyone in your household ever been diagnosed with leishmaniasis?**	Education	Bachelor Enrolled (4th / 5th year of degree)	6.6	21.3	496.024	0.000
		Doctorate	3.4	5.9		
		Master	4.3	21.9		
		Diploma / Intermediate	8.7	5.9		
		Bachelor	4.3	14.1		
		Post Doctorate	1.0	2.6		
**Are there measures taken in your community to control sandfly population?**	Education	Bachelor Enrolled (4th / 5th year of degree)	14.5	13.4	303.177	0.000
		Doctorate	3.6	5.7		
		Master	14.0	12.1		
		Diploma / Intermediate	12.2	2.4		
		Bachelor	10.2	8.2		

		Post Doctorate	1.9	1.8		
**Do you use bed nets daily?**	Education	Bachelor Enrolled (4th / 5th year of degree)	11.0	16.9	344.363	0.000
		Doctorate	2.8	6.5		
		Master	10.5	15.6		
		Diploma / Intermediate	10.8	3.8		
		Bachelor	9.4	9.0		
		Post Doctorate	1.2	2.4		
**Do you think having knowledge about One Health can help in preventing leishmaniasis?**	Education	Bachelor Enrolled (4th / 5th year of degree)	20.4	1.7	45.168	0.000
		Doctorate	7.0	0.9		
		Master	20.6	1.2		
		Diploma / Intermediate	10.7	0.9		
		Bachelor	13.4	1.2		
		Post Doctorate	2.5	0.1		
**Have you implemented any preventive measures in your home or community based on your knowledge of One Health?**	Education	Bachelor Enrolled (4th / 5th year of degree)	21.1	6.8	139.016	0.000
		Doctorate	6.5	2.9		
		Master	19.4	6.7		

		Diploma / Intermediate	12.8	1.8		
		Bachelor	14.8	3.5		
		Post Doctorate	1.9	1.8		
**Are there any community initiatives or programs promoting One Health concepts in your area?**	Education	Bachelor Enrolled (4th / 5th year of degree)	10.6	17.2	248.567	0.000
		Doctorate	3.8	5.6		
		Master	8.4	17.7		
		Diploma / Intermediate	9.3	5.3		
		Bachelor	9.4	9.0		
		Post Doctorate	1.0	2.6		
**If 1, are you involved in any of these initiatives?**	Education	Bachelor Enrolled (4th / 5th year of degree)	7.3	20.6	300.372	0.000
		Doctorate	3.4	5.9		
		Master	7.5	18.6		
		Diploma / Intermediate	8.9	5.7		
		Bachelor	6.3	12.1		
		Post Doctorate	0.8	2.8		

28.
29. S. Table 7: Association of knowledge, attitude, and perception with the demographic variable workplace of participants.

Question	Variable	Unique Variable	+Ve Knowledge %	-Ve Knowledge	Chi-square	Significance level (p-value)
**Have you heard about Leishmania?**	Workplace	Research Institute	5.3	0.0	34.722	0.000
		University	16.0	0.6		
		Government Hospital	31.4	1.0		
		Private Sector	9.0	0.0		
		Private Hospital	5.1	0.0		
		College	0.9	0.0		
		N/A	29.8	0.8		
**What is the causative agent of Leishmania?**	Workplace	Research Institute	4.7	0.6	415.677	0.000
		University	15.4	1.2		
		Government Hospital	28.2	4.2		
		Private Sector	8.3	0.7		
		Private Hospital	4.6	0.5		
		College	0.9	0.00		
		N/A	23.8	6.9		
**Is Leishmaniasis an infectious disease?**	Workplace	Research Institute	2.0	3.0	123.956	0.000
		University	5.5	10.2		

		Government Hospital	15.4	14.2		
		Private Sector	4.3	4.0		
		Private Hospital	2.9	2.0		
		College	0.3	0.6		
		N/A	12.1	15.6		
**Is Leishmania contagious?**	Workplace	Research Institute	2.0	3.0	123.422	0.000
		University	5.5	10.2		
		Government Hospital	15.5	14.2		
		Private Sector	4.3	4.0		
		Private Hospital	2.9	2.0		
		College	0.3	0.6		
		N/A	12.1	15.6		
**Do you hear about mortality from leishmania?**	Workplace	Research Institute	2.6	2.6	199.535	0.000
		University	8.9	7.0		
		Government Hospital	17.6	10.8		
		Private Sector	4.3	4.0		
		Private Hospital	3.8	1.3		

		College	0.9	0.0		
		N/A	16.9	11.1		
**Is leishmania transmitted from human to human?**	Workplace	Research Institute	2.9	1.8	195.037	0.000
		University	7.5	7.2		
		Government Hospital	14.0	13.4		
		Private Sector	4.3	3.4		
		Private Hospital	1.6	2.0		
		College	0.3	0.6		
		N/A	17.5	11.0		
**Is leishmania transmitted from animal to animal?**	Workplace	Research Institute	3.6	1.1	98.328	0.000
		University	10.5	4.7		
		Government Hospital	19.6	9.6		
		Private Sector	6.1	1.3		
		Private Hospital	3.4	1.1		
		College	0.6	0.3		
		N/A	21.2	6.7		
**Is leishmania transmitted from animal to human?**	Workplace	Research Institute	4.3	0.8	179.072	0.000
		University	11.4	4.2		

		Government Hospital	24.5	4.8		
		Private Sector	6.9	1.4		
		Private Hospital	4.3	0.8		
		College	0.3	0.3		
		N/A	20.2	8.0		
**Is leishmania transmitted from human to animal?**	Workplace	Research Institute	2.4	2.3	249.218	0.000
		University	7.4	7.2		
		Government Hospital	10.2	12.5		
		Private Sector	3.7	4.3		
		Private Hospital	1.7	1.6		
		College	0.0	0.6		
		N/A	13.4	11.3		
**Does leishmania transmit from pregnant women to their offspring?**	Workplace	Research Institute	2.4	2.1	136.214	0.000
		University	7.1	7.0		
		Government Hospital	17.2	9.5		
		Private Sector	4.2	2.9		
		Private Hospital	2.9	1.3		

		College	0.0	0.6		
		N/A	17.2	8.2		
**Does leishmania transmit from infected animals meat or milk?**	Workplace	Research Institute	1.9	2.5	189.063	0.000
		University	3.2	10.8		
		Government Hospital	9.1	16.6		
		Private Sector	3.5	3.3		
		Private Hospital	1.1	2.4		
		College	0.3	0.3		
		N/A	11.2	14.6		
**Have you ever heard about leishmania treatment?**	Workplace	Research Institute	5.0	0.2	225.156	0.000
		University	15.0	1.6		
		Government Hospital	27.9	1.4		
		Private Sector	7.5	0.8		
		Private Hospital	4.8	0.3		
		College	0.9	0.0		
		N/A	26.5	3.2		
**If 1, what treatment is done for**	Workplace	Research Institute	4.7	0.6	387.102	0.000
		University	14.2	2.4		

**Leishmania infection?**		Government Hospital	27.3	5.1		
		Private Sector	7.0	2.4		
		Private Hospital	3.9	1.2		
		College	0.6	0.3		
		N/A	21.8	8.9		
**Have you ever heard of the term "One Health"?**	Workplace	Research Institute	4.7	0.5	214.368	0.000
		University	16.3	0.3		
		Government Hospital	27.3	5.1		
		Private Sector	9.0	0.0		
		Private Hospital	4.4	0.7		
		College	0.6	0.3		
		N/A	27.8	2.9		
**Have you received any formal education or training on One Health concepts?**	Workplace	Research Institute	1.9	3.4	92.068	0.000
		University	9.7	6.9		
		Government Hospital	16.2	16.2		
		Private Sector	5.4	3.6		
		Private Hospital	3.5	1.6		

		College	0.3	0.6		
		N/A	16.6	14.1		
**Do you Know Zoonotic?**	Workplace	Research Institute	5.3	0.0	90.698	0.000
		University	16.6	0.0		
		Government Hospital	31.7	1.7		
		Private Sector	8.8	0.2		
		Private Hospital	4.8	0.3		
		College	0.9	0.0		
		N/A	28.4	2.2		
**Is leishmania a Zoonotic disease?**	Workplace	Research Institute	5.0	0.2	111.443	0.000
		University	15.3	1.3		
		Government Hospital	29.3	3.1		
		Private Sector	8.5	0.6		
		Private Hospital	3.9	1.2		
		College	4.0	1.2		
		N/A	26.4	4.3		
**Have you or anyone in your household ever been**	Workplace	Research Institute	0.6	4.7	152.866	0.00
		University	4.5	12.0		

**diagnosed with leishmaniasis?**		Government Hospital	10.1	22.3		
		Private Sector	1.1	8.0		
		Private Hospital	2.0	3.1		
		College	0.0	0.9		
		N/A	10.0	20.7		
**Are there measures taken in your community to control sandfly population?**	Workplace	Research Institute	2.5	2.8	171.574	0.000
		University	8.2	8.3		
		Government Hospital	19.3	13.1		
		Private Sector	4.6	4.4		
		Private Hospital	4.7	0.5		
		College	0.3	0.6		
		N/A	16.8	13.9		
**Do you use bed nets daily?**	Workplace	Research Institute	2.8	2.5	358.610	0.000
		University	4.4	12.2		
		Government Hospital	17.1	15.3		
		Private Sector	5.1	3.9		
		Private Hospital	4.1	1.0		

		College	0.6	0.3		
		N/A	11.6	19.0		
**Do you think having knowledge about One Health can help in preventing leishmaniasis?**	Workplace	Research Institute	4.2	0.0	151.960	0.000
		University	13.4	0.3		
		Government Hospital	23.8	2.6		
		Private Sector	6.1	0.7		
		Private Hospital	3.6	0.3		
		College	0.3	0.3		
		N/A	23.2	1.7		
**Have you implemented any preventive measures in your home or community based on your knowledge of One Health?**	Workplace	Research Institute	3.6	1.7	59.767	0.000
		University	11.4	5.2		
		Government Hospital	25.4	7.0		
		Private Sector	7.1	1.9		
		Private Hospital	4.4	0.8		
		College	0.6	0.3		
		N/A	24.1	6.6		
**Are there any community initiatives or programs promoting**	Workplace	Research Institute	1.8	3.4	201.495	0.000
		University	5.9	10.7		

**One Health concepts in your area?**		Government Hospital	16.0	16.4		
		Private Sector	4.3	4.7		
		Private Hospital	3.5	1.6		
		College	0.0	0.9		
		N/A	11.0	19.7		
**If 1, are you involved in any of these initiatives?**	Workplace	Research Institute	1.6	3.7	244.381	0.000
		University	4.9	11.6		
		Government Hospital	12.6	19.8		
		Private Sector	3.7	5.3		
		Private Hospital	3.5	1.6		
		College	0.0	0.9		
		N/A	7.9	22.7		
